# Exploring Chinese and Ethiopian higher VET adolescent learning motivation through the lens of self‑determination theory

**DOI:** 10.1371/journal.pone.0285439

**Published:** 2023-05-30

**Authors:** Sabika Khalid, Chunhai Gao, Cai Lianyu, Jiang Lu, Lu Xiuyu, Endale Tadesse

**Affiliations:** 1 College of Teacher Education, Zhejiang Normal University, Jinhua, Zhejiang Province, China; 2 Faculty of Education, Shenzhen University, Shenzhen, China; Ahvaz Jundishapur University: Ahvaz Jondishapour University of Medical Sciences, ISLAMIC REPUBLIC OF IRAN

## Abstract

Ethiopia and China share a common educational agenda in cultivating and obtaining competent vocational graduates who fulfill the need of the modern, technologically advanced industrial workplace. Unlike most evidence, the present study adopted Self-determination Theory to understand Ethiopian and Chinese higher Vocational education and Training (VET) college students’ learning motivation. Hence, this study recruited and interviewed 10 volunteers senior higher VET students from each setting to unfold their satisfaction with psychological needs. The study’s main finding affirms that although both groups felt the autonomy of choosing the vocational field they sought to master, their learning process was submissive to their henpecked teaching method, which ultimately restrained the participants’ feeling of competence for being enclosed in less practical training space. As per the study findings, we forward feasible policy and practical implications suggested for meeting the motivational needs of VET students and promoting learning stability.

## Introduction

It is well documented that student learning motivation substantially determines their learning satisfaction, engagement, competency, achievement, and performance. Those who exhibited surplus learning motivation leverage their learning outcomes, such as test scores and GPA, and alleviated dropouts [1–6]. Hence, it became desirable among scholars to unfold determinants of student motivation that considerably indicate what makes learning exciting and meaningful and strategies that foster the development of motivation [6,7–9]. Although a large body of evidence examined student motivation toward their study and identified motivating and demotivating factors, a bulk of the literature is concentrated in primary, secondary, and higher education (universities) [e., 10]. The present study intended to shed light on the learning motivation of Vocational Education and Training (VET) college students who are situated in a profound learning experience that involves more technical knowledge and skills than theoretical compared to primary, secondary, or university students [11,12]. VET students have a higher likelihood of joining the workforce, so pursuing their VET in a monotonous program, not manifesting solid vocational skills, and diminished motivation may follow, likely leading to low competency, performance, and high dropouts [13–16]. Thus, identifying motivational and demotivation factors of VET students illuminate policymakers, educators, and non-government organizations with robust strategies that would be an intervention that boosts the motivation of VET students that most developing countries VET lack to obtain due dearth of evidence [12,14], while the African Union and United Nations announced the development of VET is one of the top priorities to be considered [17–21]. Explicitly, this study explores the learning motivation of VET students in Ethiopia from East Africa and China from East Asia, where studies stipulated that VET students are dropping out from the program, possess lower learning engagement, competency, and performance after joining the workforce [11,19,22–25]. The current study underpins self-determination theory (SDT) from other motivation theories since the view is concerned with the motivation behind their field of choice, focusing on the degree to which an individual’s behavior is self-determined, and their SD motivation is a paramount framework in work-related research, influencing performance, satisfaction, commitment, and fostering competency [7,15,26–28]. Besides understanding the application of SDT as an approach to exploring VET students learning motivation, the present study has supplementary significance by adding literature regarding SDT in non-western countries context [c.f. 10].

## Literature review

### Self-determination theory

The present study illustrates Ethiopian and Chinese VET college students’ learning motivation factors through the theoretical lenses of self-determination theory [2,29,30] (Deci and Ryan 1985, 2000, 2014), which is rarely used in the VET setting. The STD states that individuals require rigor psychological needs and an ideal learning environment as the precondition for their well-being and development of motivation [5,8–10,23,27]. The pioneers of the theory, Deci and Ryan [2,29], indicated that SDT possesses three factors that illustrate the learning motivation of individuals that reflects individual and environmental perspectives. According to Deci and Ryan, students seek the actual psychological state of *Autonomous* that allows students to have the feeling of self-decision on the choice of learning or working; *Competency* is the second state of persuasion individuals’ need for experiencing mastery of performance; and *Relatedness* which brings a feeling of social interaction with the environment need to be satisfied to stimulate learners’ motivation [2,3,7,27]. Hence, altogether the SDT affirms that individuals’ motivations emerge from the ultimate feeling about themselves, others, and the task they opted to be engaged in [6,9,12,14,26]. Although, to the best of our knowledge, no qualitative study aims to unfold the motivation of VET college students through the theoretical perceptive of SDT, this study explains how this theory is considerably relevant to attain the principal purpose of the study. Given unlike general education, VET college students’ learning environment is markedly different, which primarily empowers adolescents to early to join the middle and low-job title (blue-collar) market with lifelong vocational skills and knowledge [11,17,18,20,31,32]. Vocational-related self-determination skills are self-supporting for vocational skill accommodation, resolving problems in the workplace, accomplishing learning tasks, and developing future work plans [15,33].

Thus, compared to general education students, it is creditable to explore VET college students’ motivation during their study by investigating the value of *autonomy* to understand the need for autonomy support from the teacher on their learning process and the choice of field of study from their parents [8–10,15,34]. A shred of prior evidence demonstrated that if teachers administer their teaching-learning process by question and answer, it leads to directive learning through scoped teaching curriculum, which promotes controlled learning that mitigates the opportunity of autonomous learning, which has an adverse effect on their learning motivation [3,5,15,27]. Moreover, Reeve asserted that student learning motivation should be enhanced by avoiding a controlling way of teaching, which does not make learning meaningful and exciting for students as long as they are not a part of and in charge of it [35]. In the meantime, it has to be noted that VET college students ought to obtain decent autonomy-supportive parents who appreciate and sympathize with the field choice of their children for selecting a field of study in which they are passionate and inquisitive on it [9,12,23], which earlier literature is suggested to be investigated from the Chinese culture "filial piety" ideology [10,31,34]—following that the SDT claims that students develop their motivation as long as they feel effective in interacting with the environment through obtaining up-to-date competencies [5,11,27,34]. A preceding study that assessed high school students’ motivation for quitting music study shown that students could not obtain the intended skill and knowledge they expected to become competent due to the weak lesson plan of the teacher that had no room for new skills [26]. Evans added that students lose the passion for pursuing their learning when they believe their skill and ability are limited and insufficient to solve problems. A recent study in Finland on 62 music students who participated in workshops found that students perceived that they had significantly developed their competency due to the autonomy-supporting learning environment they got [8]. A recent cross-sectional study that recruited large-scale higher VET college students in several provinces of China donated that due to the teaching practice lacks (work) project-based learning or teacher-centered teaching, students lose their motivation throughout their program [11,12,36–39] which promotes "devocationalism" [10,11,39], and "false consciousness" [40]. Likewise, a longitudinal survey in Shaanxi and Zhejiang province VET schools suggested that students are highly demotivation enough to drop out due to low achievement and parents losing interest in their children being in VET colleges [10,37,41,42] unless the field they are studying has a decent possibility for further education [31,32].

Meanwhile, it is noteworthy that unless students feel autonomous in their learning process, experiencing competency will not maintain once intrinsic motivation for learning [2,4,9,34]. The third component of SDT is relatedness, considered the iconic external motivational factor relevant to VET college students’ learning process and motivation [4]. They are called to possess solid and active social interaction during their study with their classmates, teachers, peers, and family, which is also interrelated with developing an autonomy-supportive environment [2,8,15]. A quasi-experimental study that administered the survey and focused group discussion among a 3-year bachelor Dutch law university students who are in traditional teaching and problem-based learning class to compare their STD claimed that students in problem-based learning environment showed an exceeding behavior of motivation towards their course than their counterparts in lecture-based class since they built a solid social circle and communication with peers and teachers through the tutorial and non-formal academic relationship the program facilitates for them [5].

### VET in China

**Fig 1:**
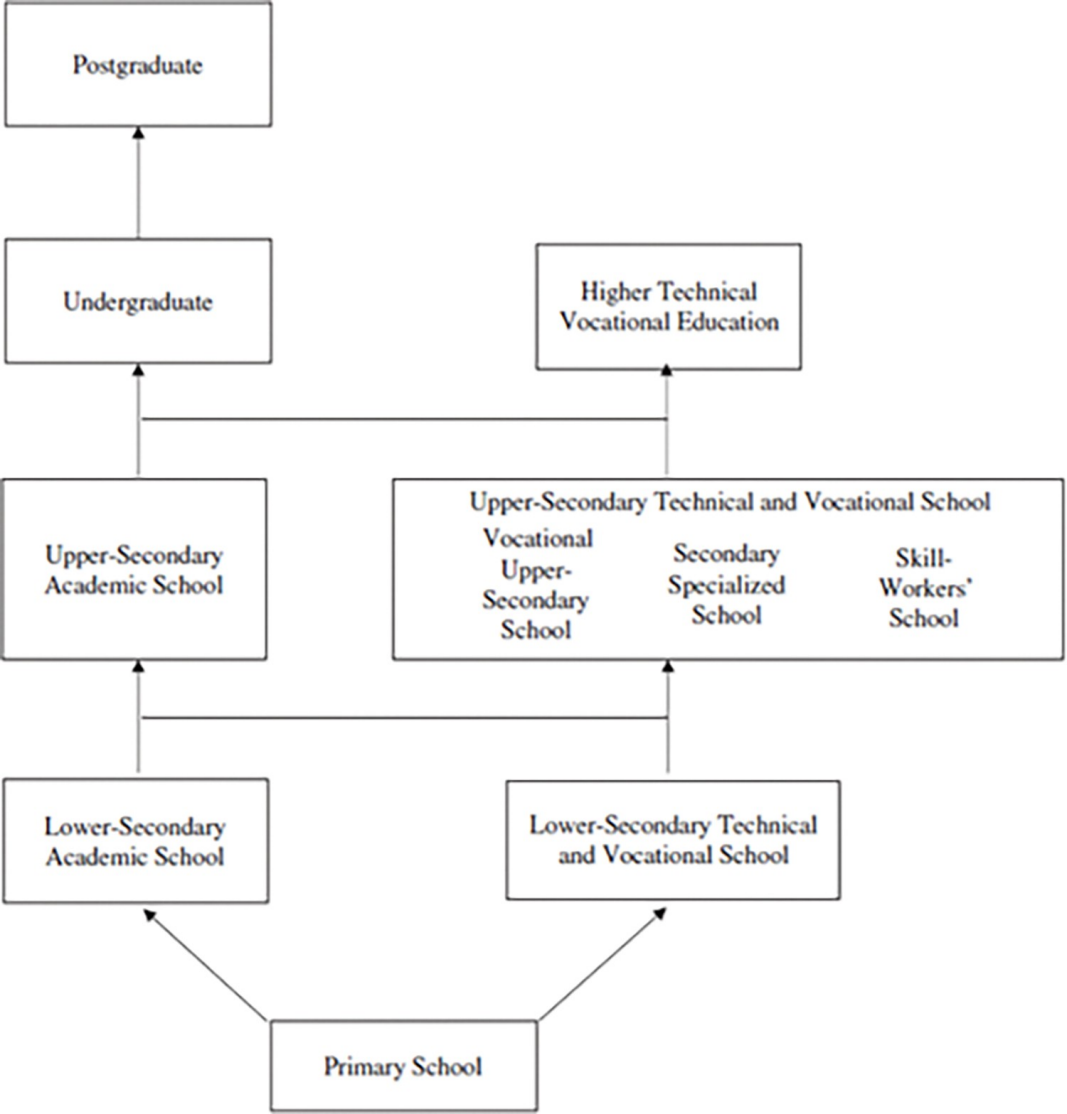
The education system in China.

Like other countries [39], the Chinese government expand access to VET across the nation not only this particular education sector has a significant contribution to the short- and long-term economic development through giving out productive and young human capital, and diminishing unemployment [11,13,23,32,42–45], but also to provide the source of genuine experts who cherish their profession [23,36,38]. Although scholars and policymakers are striving to foster the VET quality and rate of return [18,45], still the society, students, and parents do not endorse its privilege [4,22,36,39,42,44]. Unfortunately, industrial products of China labeled as "Made in China" as a stereotype of low-quality merchandise around the globe [13], which became an inspiration for the government to launch "Made in China 2025" to transform the industrial products through competent VET graduate workforce [23,40]. Speaking of which, according to the China educational structure, VET is set in three academic levels, which are primary (1–6), lower-secondary (7–9), upper-secondary (10–12), and higher VET (3) years [12,13,18,22,36,46,47] (See. Fig 1). After completing the upper-secondary level, students are required to sit for the university entrance examination (Gaokao) to determine their ultimate end to be in Higher education institutions or Higher technical and vocational training colleges [10,23,39,43,47], which is the sole means to climb the social class ladder [32,39], due to the meritocracy system [37,40,42]. A national report stated that higher VET enrollment had shown a sizable increment from 2010 of 9.62 million to 12.87 million in 2019, a difference of 3.14 million. Particularly, the recent enrollment of higher VET students in 2018 and 2019 has shown an enrollment growth of 1.47 million, with a 12.97% growth rate [10,28,45,47]; nevertheless, the teacher-student ratio is low [11,43]. For decades higher VET colleges have been entitled to the last resort for academically incompetent [13,31,37,39,42,46], low socioeconomic, and rural adolescents whose parents demand them to their financial support soon [36,42], which initiate unhealthy teachers’ stereotypical [39,40], high dropout, and low retention [23,41,45], and shortage of skilled labor [11,18,23,33,46]. Thus, it is considered worthy and relevant to assess the higher VET learning environment that recourse millions of students who are accounted for the valuable, necessary competent human capital that determine the genuine development of the nation [23,36,43]. As a result, the government is striving to promote the VET through financial, physical, and human resources and persuading the advantage of VET the opening door for early employment [28,42,48], although the benefit reduces through time [42,48,49]. Despite the efforts, the higher VET could not produce adequate competent graduates due to an immature curriculum and learning environment, which possess outdated facilities that depress the learning motivation of students [11,33,36,40], which results for non-involved, a non-engaged, non-competent graduate that unseemly for the modern technological, the industrial employee need [33,45–48]. Moreover, it has been subsequently addressed that alongside the barren teaching practice, which does not involve real-life learning process through enterprises, the teachers forsake practical competency; thereby, students acquire "vocational" skills beyond theoretical knowledge [4,10,11,13,36,38,42,44], which calls for "dual-qualified" teachers [43,47].

### VET in ethiopia

In recent years, the Ethiopia government has expanded access to VET institutions across the country, which has resulted in a substantial increment of enrollment, explicitly female students [50–52]; however, dropout is evident due to its opportunity cost [17,25]. As the country is under an effective strategy that aims to become a transformational middle-income country through the industry-led plan [21,25,53–55], it is evident that VET is the entire educational sector which contributes in producing competent workforce which gratifies the need of 21^st^ century technological equipped industries [19,52,56], through competent-based training [20,21,50,51]. According to the Ethiopian educational structure, primary education consists of eight years made up of 4 years (1–4) of the first and (5–8) second cycle of primary school, two years (9 and 10) of the first cycle secondary education, two years (11 and 12) of second-cycle of secondary, and higher education or universities [25,52]. As alike as China, Ethiopian students are expected to obtain the minimum of cutting-edge national examinations at secondary and preparatory education to join higher education or universities; else, VET institutions procure failed students in different vocational fields of study so that they can occupy the demand of workforce in the blue-collar job given by national and international enterprises [21,24,51,55–57], which spread the stereotype of being a VET college student as a failure in life [52,58], result demotivation [57]. Surprisingly, a prior study that examined second-cycle of secondary school (11 and 12) students’ perception of VET noted that most participants have a positive attitude towards VET [17]. On the other hand, based on the recent national survey of VET graduate’s employers, enterprises are facing an excessive challenge in finding competent graduate who is familiar with current technological equipment and committed to their job [19,20,51,52,54,55,57]. Tamrat has asserted in his book that the country is losing the attraction of international investments due to the lack of skillful and competent VET graduates [52]. A consecutive body of studies in Ethiopia confirmed that VET colleges possess unqualified and incompetent teachers who do not acquire not only deep theoretical knowledge but also solid industrial and business experience [e.g., 52,55,58], and unfeasible curriculum which is incapable of delivering the essential competency [57]. Meanwhile, the government proposed a roadmap for education development (2018–2030) which affirms that VET the primary concern of matter in fostering the policy, management, curriculum, and resources [57], which promotes a learning environment of 30% to the classroom, and 70% field or workplace learning cooperated with the government and private institutions [19,21,58], nonetheless VET institutions don’t have adequate resources to provide minimum knowledge students couldn’t expand it in the work place [50,54,58], and private enterprises are reluctant to bring VET college students into their working place [53,55], which leads to demotivated students and incompetent graduate [20,24], and unemployment [51,52,54,56]. Latter evidence involving 1398 VET college students in Ethiopia from textile and garment-related departments revealed that students who aspire to open their businesses or be entrepreneurs with the skill they learn are motivated and satisfied with their learning experience [21]. Hence, it is evident that exploring VET college students’ motivation and determining the factors through SDT to unfold why students exert effort to accomplish the goal and purpose of studying VET. What are the reasons that led to the attempt to achieve this goal? This allows comparable studies to identify the key motivational factors for VET students learning motivation [C.f. 12].

## Method

### Research design

The current student adopted a comparative narrative qualitative research approach comprising ten senior (graduand) students from Chinese and Ethiopian higher VET colleges located in Shenzhen and Addis Ababa, respectively. It is evident that underpinning epistemological assumption, a paradigm that unfolds individuals’ experiences, perspectives, and feelings about their environment through interaction with it, is substantially outfitted to understand motivation [15,59]. Therefore, we conducted a semi-structured interview with the two groups of participants and constructed and analyzed deductive thematically or theory-driven analysis.

### Participants

The present qualitative study aims to explore VET college students’ motivation through the SDT perspective, articulating the comprehensive motivational factors that vehicle extensive vocational skill learning and prepare a productive workforce. Initially, we recruited voluntary students to participate in the study from higher VET colleges from Shenzhen and Addis Ababa cities in China and Ethiopia, which are known for possessing passable TVET colleges with rich department diversity. Subsequently, after we obtained ethical approval from the Ethics Committee of Shenzhen University, we visited higher TVET colleges in both cities to handpick senior volunteer students from distinct departments, and written consent was obtained from participants. As is presented in Table 1, we obtained 10 senior higher VET college students each from both settings made up of a total of 20 participants aged 21 to 23 years with no parental educational level higher than secondary education, which confirms who often join VET sector [36,48].

**Table 1:** Participants’ personal and academic information.

Participants	Gender	Age	Field of Study	Mother Educational Level	Father Educational Level
ETH1	Male	21	Budget service	No Education	No Education
ETH2	Male	21	Accounting	No Education	No Education
ETH3	Female	22	Tourism Management	Primary Education	Senior High school
ETH4	Female	22	Nursing	Junior High school	N/A
ETH5	Male	23	Marketing	N/A	Vocational Diploma
ETH6	Male	22	IT	Primary Education	Bachelor Degree
ETH7	Female	21	IT	Primary Education	Primary Education
ETH8	Female	23	Hotel management	Junior High school	Junior High school
ETH9	Male	22	Automotive	No Education	Vocational Diploma
ETH10	Male	22	Accounting	Primary education	Primary education
CHN1	Female	21	Legal affairs	Junior high school	Junior high school
CHN2	Male	22	Food inspection and testing technology	Primary Education	Primary Education
CHN3	Female	21	Modern logistics management	junior high school	Senior high school
CHN4	Male	22	Finance	No Education	junior high school
CHN5	Male	23	Material engineering technology	primary school education	primary school education
CHN6	Male	22	Financial technology	primary school education	primary school education
CHN7	Male	22	Analysis and inspection technology	junior high school	junior high school
CHN8	Male	23	legal affairs	primary school education	junior high school
CHN9	Male	22	modern logistics management	No education	No education
CHN10	Female	23	Fintech application	junior high school	junior high school

### Data gathering procedure and data analysis

The present study performed a Face-to-face semi-structured interview, enabling us to acquire factual information on the participants’ experiences [20]. The semi-structured interview questions were self-determined theory and literature review driven. “Do you take an interest in the field you study outside studying hours?”, “What was important in your choice?”, “What emotions arise relating to your study? Why? To what extent do you feel stressed at studying? were the sample interview questions. The interview protocol was constructed in English; later, with the help of professional translation, it was translated into Amharic and Chinese (Mandarin) language to enable the respondents to share their accounts in their language and from their point of view. The first author is a native Ethiopian scholar, while the third author is a native Chinese academic and psychologist who administered the interview, which hardly took 25 to 45 minutes. During the interview, the authors performed the probing questing tactic to ensure the participants’ accounts’ reliability and accuracy, which enabled the interviewer to throw light on obscure responses and get clarification of the participants’ accounts. Moreover, after the interview, we translated the transcription into English and used the deductive thematic approach that pronounces the data coding into predetermined themes.

After a professional translational of each interview transcription into English, NVivo 12 software was used to administer a deductive thematic analysis by creating nodes with the sub-nodes under the preconceived themes. The study used a self-determination theoretical framework; therefore, interpretation-focused coding techniques were adopted to develop codes. Moreover, the trustworthiness and validity of the data analysis and interpretation were enhanced by consulting with native scholars. We developed codes and themes, checked developing interpretations against alternativeness, and sought divergent cases in the data [60]. Since the data quality depends on the connection between the researchers and participants, we established trust and privacy to ensure quality data collection and appropriate social processes.

### Research findings

After administering a deductive thematic analysis which allows acquiring of the predetermining themes accordingly to the psychological needs described by SDT [2,29,30], which unfolds the experience of Chinese and Ethiopian students learning motivation in VET colleges through the needs for *autonomy*, *competence*, and *social-relatedness*.

### Autonomy

Both groups of participants were asked about their freedom of choice in selecting the major they are interested in studying at VET college. Most respondents in both parties claimed they were experiencing independence in choosing the major and the satisfaction it creates in their learning motivation. One of the Chinese participants explained:

*I Make my own decisions in picking the major I am studying now. Because first of all, for example, a career plan you choose in the future is what kind of career you are engaged in. Of course, if you can, you should decide according to an idea in your heart. It must be excellent for you to devote yourself and work hard in this profession in the future. Then we will improve our work efficiency, as well as our self-satisfaction and realization of our value, which will be much better in this respect. (CHN4)*

Likewise, although all Ethiopian counterparts have shared that they chose the career they sought to study independently, respondents also affirm that the primary reason for selecting the vocational career is the extensive need for the workforce in the job market. They aimed to study that field to optimize the chance of obtaining a decent job after graduation.

*I chose the field of hotel management instead of other fields which I am interested in since, in Ethiopia, the hotel and tourism business is boosting year to year, which enhances the construction of hotels around the country which seeks several vocational college graduates…………so, that makes me energetic and interested to learn seriously to obtain a job in five-star hotels. (ETH8)*

Even though the interview with Chinese and Ethiopian VET college students illuminates that they had free will to choose their vocational major to learn through their interest and long-term goal, the participants experience no supportive autonomy from the teacher. The interviewee mentioned that they had yet to feel the satisfaction of autonomy from the teachers in the teaching-learning process as the curriculum is rigid, and traditional teaching methodology needs a very structured daily routine with little room for change. CHN2 expressed:

*I think the teacher controls our study because the teachers possess a large credit of courses, and then, the number of pages of the course book is more. Then we have to complete it……. so we feel less learning freedom…. have little time for what we need, and have extracurricular time to learn what we want to learn, so there is free time to learn.*

Likewise, the Ethiopian interviewee added the same experience of an unsupportive autonomy learning environment excreted by the teacher. The students experience authoritarian teachers who get angry or offended for getting a suggestion from students or comments regarding their learning process, which makes them feel apathetic about their learning.

*Many days we go to school while the teacher is unavailable, or we get so annoyed by multiple times going to school, but other classes have occupied the computer lab………. We tried to talk to the teacher or management to fix this problem by the solution we thought………unfortunately, the teachers got aggressive with us for being genuine about our study, which created a harsh learning environment. (ETH3)*

Generally speaking, although students perceive the autonomy of choosing the vocational field they sought to master, neither Ethiopian nor Chinese interviewees have experienced autonomy in their learning process with the teacher. They stipulate that their satisfaction with the teachers’ low supportive autonomy ranged between these ends. The need for choice and autonomy was expressed along with a preference for a stable structure and/or a person that can offer advice. Structure functions as a facilitator were not fulfilling the need for autonomy. Participants preferred to be given a few options instead of having an undefined range of possibilities [15].

### Competency

The Ethiopian and Chinese VET students must be more competent in vocational mastery. Throughout the three years they stayed in the VET college; they did not believe they developed confidence in their field and felt professional. In a vocational school, students are required to handle competence by exposing them more to practical or vocational workplaces; nevertheless, there is no considerable learning environment where students cherish a feeling of competence by exposing them to innovative and up-to-date knowledge.

*We have more classes and less actual operation or practice. That is why the teacher mostly finishes the course by talking about the theory class; I will feel empty and lack practical support. . . . sometimes When I go into the operation, I will find that what he said is very simple. But always listen to the theory; it will feel empty and does not sound like that strong interest. I want to operate with my hands. (CHN7)*

According to our participants from both sides, it is evident that students would not experience the feeling of confidence in their competency in the workplace after graduation. Students noted that they could not handle situations in the workplace. However, the school organizes multiple internship chances in different enterprises, which allows students to foster their practical skills other than in the school workshop.

*Getting institutions willing to take internships was a serious problem since there is no formal way for the school to collaborate with institutions for different majors……. Although after much suffering, I went to the internship, I felt frustrated and awkward in the workplace since I did not have sufficient knowledge and skills to cope. (ETH4)*

*The teacher takes us to the workshop to use machines to learn. However, the number of people in the class is extensive and limited equipment; the teacher cannot be unified demonstration and can only group, divide into two groups and then around the teacher. During the teacher’s demonstration and explanation, students standing at the back cannot see or even hear the teacher a comprehensive explanation. (CHN2)*

In addition, most of the students’ responses show that since the teachers exhibit unsupportive autonomy indicators during the teaching-learning process, it causes an adverse effect on their need for satisfaction of competency [8].

*Well, most of our class is more inclined to lecture because the curriculum has no room for our learning needs or suggestions, so the teacher teaches his/her class according to his book and then feels very bored. (CHN3)*

*Sometimes the teacher wants to show us things in the computer lab. However, we mostly find the ICT lap occupied, or the ICT lab may not be available during working time, and nobody says anything…… so many of us feel fear of graduating without knowing the real knowledge, so we mostly go to the private institute who teach those software programs we need them in the job place. (ETH2)*

The interviewee from Chinese and Ethiopian VET college considers that the fundamental aim of TVET, and being in, is to furnish students with a particular set of technical skills that leads to graduates who feel competent and "vocationalism" to their corresponding job. In this respect, our respondents assert that all the mediums (classroom teaching, workshop, internships) intended to empower students with solid technical knowledge and skill were absent. The participants remarked that teachers constantly use a teacher-centered approach that supports the delivery of instruction to large classes because they are considered cost-effective in developing countries’ education systems. The experience and view of our participant’s adherence to [20] that due to the extensive practice of theoretical-based or teacher-centered teaching methods, students get passive in the learning and run out of motivation. Finally, they graduate and join the workforce without acquiring solid vocational knowledge and skill [15].

### Social-relatedness

Our participants have mentioned their experience with the satisfaction of the relatedness from the two frequently mentioned perspective themes. The first social relatedness vocational students experience is with their classmates and peers, which significantly determines their satisfaction in terms of the communication, social life, and relationship they perceive. Chinese VET students explicitly showed robust relatedness with their classmates and peers through several formal and non-formal groups they made.

*Many students are very good for me because the class has a different committee for different purposes……. also organizes some activities. Then the class committee will meet, then the others will know some. (CHN3)*

*I positively interact with my classmates, and our relationship involves professional knowledge sharing……; I think I can better understand some knowledge and have a better teacher to communicate….I feel happy talking to friends… hanging out with them outside the classroom to play ping-pong, basketball, or games; you can talk with friends, mentors, and helpful friends to increase your knowledge of society. (CHN9)*

Even though both Chinese and Ethiopian VET participants came from different areas of the country, unlike Ethiopian VET students, most Chinese students experienced a splendid relatedness with their classmates. School peers for academic and non-academic purposes; since all VET students in China stay in the dormitory the school provides, they have enormous time to get along with their classmates and peers after class.

*….The teacher insists we create a small group of 5 members intended to create a group feeling that helps each other…….. (ETH2)*

*….We have different social media groups with classmates and teachers, which made us keep close and updated with each other… (ETH8)*

*…We have a group, but we do not have that much understanding among us, which may enhance our learning, because everyone leaves school after class, and just talking in the group we made on social media is not enough to create that bond among us…(ETH3)*

Simultaneously, the participants shared the relatedness they experienced with their teachers. Likewise, Chinese VET students encounter promising kinship with their teachers and feel that their teachers are easygoing or approachable to discuss.

*I have a good relationship with my teachers. I believe it is good to keep a smooth relationship with the teacher…… because I have an advantage by interacting with him frequently….. for example, when I study, if I want to ask something which is not clear and if I do not want or could not get help your classmates in the dormitory…. So I freely run to the teacher to ask him/her for help…..I think it’s very important. (CHN5)*

*I made a good relationship with most of the teachers, I think it is good…. on the one hand, it can promote your learning motivation and passion, on the other hand, the teacher’s words will also give you some help, such as I often communicate with the teacher, or discuss some problems. (CHN2)*

Unfortunately, when it comes to Ethiopian VET students, most of them pointed out that most of the teachers are part-timers, demotivated, and dissatisfied with their job; thus, there is no room for students to feel related to their teachers.

*Most of the excellent teachers are hired part-time……. So whenever we need to reach them out of several inquiries, they are not available….. because of that we do not have any relationship with teachers except the teaching time…..*

## Discussion and conclusion

The present qualitative study possesses a breakthrough aim that allows us first to look and learn about Ethiopian and Chinese VET students’ learning motivation through the lens of self-determination theory, which substantially determines learners’ academic engagement, competency, and performance [1,5,6,], through the theoretical lens of SDT [2,29]. The SDT describes individual motive for a particular undertaking emerges from the ultimate satisfaction the individuals experience in their learning environment of autonomy, competence, and social-relatedness [2]. Hence, to the best of our knowledge, this study is the first of its kind to shed light on two nations’ (China and Ethiopia) higher VET college students’ self-determined motivation through intensive with 10 Ethiopian and Chinese voluntary participants, which provides key implications in the striving process of fostering the productivity of VET institutions in both countries.

After performing the deductive thematic analysis, which relies on the intended theory furnished by the study, the study’s finding demonstrates that the need for autonomy of both group’s participants experience free will for choosing the major they sought to study when they joined the college. However, the finding shows that the choice of discipline depends on their intrinsically interested or extrinsically preferred as it is able them to obtain a promising earning job. It is an exciting finding explicitly to hear from Chinese participants while existing literature elucidates Chinese controlling parenting that does not allow their children to cherish parental autonomy and supportiveness [10,31,34]. The rational explanation is that apart from the preceding evidence, the current study finding presents that VET student parents were not educated enough to interfere or be involved in their children’s vocational decisions [36,48]. From the interpreted feeling and experience, we can observe that they feel satisfied with having the freedom of choice in their vocational major and have shown dedication and enthusiasm for it [9]. Moreover, besides parental autonomy supportiveness, participants discussed their experience with the need for autonomy with their teachers. The finding from the interview response demonstrates that VET teachers in both countries do not involve students in their teaching-learning process so that they can be part of it by putting their ideas or decision making. The Chinese participants frequently mentioned that the teachers stick with the course book and should finish in line with the teaching credit hour. In contrast, Ethiopian participants showed low autonomy in their learning process through poor school management and unprofessional teachers with no ear to listen to the students’ voices.

In contrast, students come to the school while their class is occupied or the teacher is absent without advance notice, which demolishes participants learning motivation. ETH4 said that "…. Imagine what we feel coming to the school by taking early morning long public bus travel to the school to learn, and the teacher is not there, or… the computer lab is closed… or. . . the other class occupies the workshop… we tried to complain on the teachers. . . instead of excusing us, he gets aggressive, and punish us for no reason". These findings are consistent with a shred of later evidence, which claimed that the advantages of teachers’ autonomy support for students’ autonomous motivation do not appear to be culture-dependent [9].

Regarding participants’ competency, the interview data analysis findings affirm that Chinese and Ethiopian participants do not state that they do not experience a feeling of confidence in their particular vocational skill after graduation. While all participants spent a minimum of three years in the higher VET college, there were supposed to show a sense of mastery in the vocational skill. Unfortunately, in line with a large volume of preceding Ethiopian and Chinese evidence [e.g., 12,37,57,58], the interview with our participants confirms that yet both higher VET teachers could not disseminate practical knowledge and skill due to their teaching method is more theoretical or bookish. The possible reason mentioned by the participants was that the colleges do not own sufficient and modern equipment in the workshop [e.g., 17,20,31,32], which explains the teacher-student ratio [11,43]. The exciting finding of this study is that students could not satisfy the need for competency in their mastery of vocational in view of the fact that initially, they do not acquire or experience the feeling of genuine autonomy support from the teacher that would entail student in the decision making regarding the teaching-learning process. In the future, we need to ask why the teachers keep doing that. Are they unqualified to disseminate practical vocational skills? Or does the curriculum and teaching environment not entertain teaching outside the box? Moreover, the experience of the Ethiopian participants verifies local literature which declares that VET institutions built an infirm collaboration with enterprises [53,55], and as a result, the student has to knock on the door of enterprise to get internship position, despite the students do not experience competency with no supervision, no feedback, and guidance.

Furthermore, the social-relatedness was the centrality of the finding shown through our participants’ social experiences and feelings. From the comparative perspective, it is evident that Chinese participants express an auspicious sense of sincere relatedness with their peers and teachers from the outlook on the soundness of the communication, relationship, and interaction experience they made for academic and social purposes. The primary reason would be the institutional structure of Chinese VET colleges that facilitate students with dormitory so that they focus on their educational goal, which indirectly fosters social interaction among students through formal groups students made by the teachers and non-formal groups students built in different amusements (sport, clubs, management). Perhaps this notable finding somehow contradicts previous evidence [39,40] regarding the bonding between teachers and students, which might brainstorm exploring more about the perception and feelings of VET teachers about their students from the angle of the social recognition VET students experience. On the contrary, participants from Ethiopian VET college pointed out that the degree of relatedness they experience and feel with their classmates is moderate since their social interaction time is during class. When the course is over, everyone goes home. In addition, students could not feel related to their teacher since teachers do not have a standard office or lounge to talk to them after class, particularly part-time teachers; do not give a social impression for the students to be easily approachable. Likewise, it has been well noted that as long as the teaching-learning process isolates students from it, there is a slim chance that both teachers and students will interact in and outside the classroom [5].

### Limitation and implication

The present study’s finding derives practical and policy implications for respective stakeholders to promote the learning motivation of VET students by identifying the potential fulfillment of the potential need. Reference to the policy implication is that both countries’ governments ought to recruit qualified VET teachers who acquire "dual qualifications." Although there is a shortage of teachers with academic and industrial or vocational knowledge credentials, the government should design and deliver short to long-term professional development according to the recruited teacher profile through intensive monitoring and evaluation. Moreover, the study suggests that both VET settings need to refine their curriculum involving external enterprises. This method of reform enhances the productivity of the learning environment for students that mainly focus on practical training than the talk-and-chalk method of teaching. Hence, students feel satisfied with the need for autonomy for having the opportunity to be part of the learning process and decision-making in real-life learning, development of competency for being in problem-solving training that promotes "vocationalism,"; and enjoy their vocational mastery, which groomed through the robust social relatedness the competency-based learning spaces built among teachers and students. Regarding the practical implication of the current study, we suggest that VET management and teachers develop a culture of smooth communication with students, who are the vital stallholders who could have contributed substantially to the curriculum’s decision-making, which primarily determines the learning motivation of students. In addition, unless adequate modern equipment which students could be interested in their learning by sensing (seeing, touching, tasting, smelling, listen), just developing a feasible curriculum and qualified teachers would be "dry firing"; thus, Ethiopia and China VET institutions are recommended to facilitate students with those vocational tools, or attract national and international industries to collaborate so that student could interact with the workplace frequently before graduation, and future job prospects.

Although this qualitative study explored the deep feeling and experiences of Chinese and Ethiopian VET students learning motivation through the lens of SDT, some limitations were inevitable. The present study sampled VET institutions from one city from both countries; thus, transferring the study’s finding to a large population setting comprising several VET institutions and cities should be considered. Moreover, the study interviewed only VET students, which made us unable to conceive word of mouth from teachers, management, and parents, so we suggest future scholars consider interviewing these stallholders that could shed light on the exotic experiences.

## Supporting information

S1 File (RAR)

S2 File (RAR)
