# Expression of Concern: Exploring Chinese and Ethiopian higher VET adolescent learning motivation through the lens of self-determination theory

**DOI:** 10.1371/journal.pone.0292445

**Published:** 2023-09-28

**Authors:** The PLOS ONE Editors

After publication of this article [1], concerns were raised about the article’s authorship, including the corresponding author designation. At this time, the *PLOS ONE* Editors have been unable to verify the information provided by the authors and consider these concerns unresolved.

During post-publication editorial review of this article, additional concerns were raised about the ethics approvals reported in the study. Specifically, the Participants subsection of the Method section states that study participants were recruited from Shenzhen, China, and Addis Ababa, Ethiopia, and that ethical approval was obtained from the Ethics Committee of Shenzhen University. The article [1] does not mention whether ethical approval was obtained from any Ethiopian institutes. During editorial follow-up, the last author confirmed that no ethics approval was obtained from Ethiopia.

In light of the unresolved concerns raised regarding authorship and ethics approval, the *PLOS ONE* Editors issue this Expression of Concern.
